# Sequencing of Single Pollen Nuclei Reveals Meiotic Recombination Events at Megabase Resolution and Circumvents Segregation Distortion Caused by Postmeiotic Processes

**DOI:** 10.3389/fpls.2017.01620

**Published:** 2017-09-26

**Authors:** Steven Dreissig, Jörg Fuchs, Axel Himmelbach, Martin Mascher, Andreas Houben

**Affiliations:** ^1^Department of Breeding Research, Leibniz Institute of Plant Genetics and Crop Plant Research (IPK) Gatersleben, Seeland, Germany; ^2^Department of Genebank, Leibniz Institute of Plant Genetics and Crop Plant Research (IPK) Gatersleben, Seeland, Germany; ^3^Domestication Genomics, Leibniz Institute of Plant Genetics and Crop Plant Research (IPK) Gatersleben, Seeland, Germany; ^4^German Centre for Integrative Biodiversity Research (iDiv) Halle-Jena-Leipzig, Leipzig, Germany

**Keywords:** single-cell genomics, pollen, meiosis, homologous recombination, crossover, crossover interference, segregation distortion

## Abstract

Meiotic recombination is a fundamental mechanism to generate novel allelic combinations which can be harnessed by breeders to achieve crop improvement. The recombination landscape of many crop species, including the major crop barley, is characterized by a dearth of recombination in 65% of the genome. In addition, segregation distortion caused by selection on genetically linked loci is a frequent and undesirable phenomenon in double haploid populations which hampers genetic mapping and breeding. Here, we present an approach to directly investigate recombination at the DNA sequence level by combining flow-sorting of haploid pollen nuclei of barley with single-cell genome sequencing. We confirm the skewed distribution of recombination events toward distal chromosomal regions at megabase resolution and show that segregation distortion is almost absent if directly measured in pollen. Furthermore, we show a bimodal distribution of inter-crossover distances, which supports the existence of two classes of crossovers which are sensitive or less sensitive to physical interference. We conclude that single pollen nuclei sequencing is an approach capable of revealing recombination patterns in the absence of segregation distortion.

## Introduction

Meiotic recombination is a key mechanism in eukaryotic reproduction which enables novel combinations of alleles and provides a mechanism for plant breeders to achieve crop improvement. Recombination patterns are shaped by genetic, epigenetic and environmental factors (Melamed-Bessudo and Levy, 2012; Mirouze et al., 2012; Yelina et al., 2012; Ziolkowski et al., 2015, 2017; Ritz et al., 2017). In many crops, including barley, recombination events occur predominantly in distal regions of the chromosomes where gene density is high. In contrast, interstitial and centromere-proximal regions containing 12–24% of the barley gene complement are marked by strongly reduced recombination rates (Baker et al., 2014). Although genetic diversity is reduced in low-recombining regions, they nevertheless contain genes and thus represent a resource that is hardly accessible to plant breeders. Therefore, significant efforts are being directed toward the manipulation of recombination frequency and distribution. Several approaches were shown to be successful, including the increase of crossovers via mutation of an anti-crossover factor (Crismani et al., 2012), epigenetic remodeling of crossover frequency via reduced DNA methylation (Melamed-Bessudo and Levy, 2012; Mirouze et al., 2012; Yelina et al., 2012; Habu et al., 2015), and shifting of crossover positions via increased or decreased temperatures (Higgins et al., 2012; Phillips et al., 2015; Martin et al., 2017). Furthermore, natural diversity of recombination patterns was shown to exist in *Arabidopsis*, maize, and *Hordeum* (Gale et al., 1970; Sall, 1990; Sall et al., 1990; Nilsson and Pelger, 1991; Sidhu et al., 2015; Ziolkowski et al., 2015, 2017).

In addition to low recombining regions limiting crop improvement, segregation distortion (SD) is another undesirable phenomenon as it reduces the chance of combining certain alleles. SD is defined as a deviation of the segregation ratio of alleles from the expected Mendelian segregation ratio. In barley double haploid (DH) populations, large proportions of the genome can show segregation distortion (Bélanger et al., 2016a). A frequent cause of segregation distortion is selection acting on genetically linked loci which results in entire chromosomal regions showing segregation distortion (hereafter termed SDR for segregation distortion region) (Hiraizumi et al., 1960; Hill and Robertson, 1966).

Taken together, tight genetic linkage of large proportions of the genome and distorted segregation resulting in a linkage drag of alleles hamper the advance of plant breeding. Future attempts to overcome these restrictions will require efficient methods to assay such effects. There are numerous methods to measure meiotic recombination in plants, including molecular markers (Salome et al., 2012), cytological visualization of crossovers (Sybenga, 1966; Anderson et al., 2003; Phillips et al., 2013), tetrad analysis (Copenhaver et al., 2000), fluorescent protein-tagged loci expressed in pollen (Yelina et al., 2013), and several pollen genotyping approaches (Drouaud and Mezard, 2011; Khademian et al., 2013; Dreissig et al., 2015). Although these methods have been successfully used to characterize recombination patterns and improve our understanding of meiosis, each of them has its specific advantages and disadvantages. The analysis of recombination by molecular markers requires the generation of a segregating population, which is laborious and very challenging for some plant species. Cytological analysis of recombination is more widespread and applicable to many plant species, yet its resolution is lower compared to sequence-based approaches and the analysis is demanding in terms of time and experience. Tetrad analysis combined with fluorescence markers is a very powerful high-throughput approach but requires the integration of reporter transgenes and is so far limited to the model species *Arabidopsis*.

Single-cell sequencing is a new technology that holds the promise to directly measure the outcome of meiosis in individual cells, e.g., microspores (Li et al., 2015) or pollen grains. We have previously developed a single pollen genotyping approach based on flow-sorting of haploid nuclei followed by whole genome amplification via multiple-displacement-amplification (MDA) of DNA and multi-locus competitive allele specific PCR (KASP) genotyping (Dreissig et al., 2015). This approach has shown the potential of single-cell analyses to measure recombination, but was limited by the number of KASP markers that could be assayed. To overcome this restriction, we took advantage of representative whole-genome amplification combined with next-generation-sequencing (NGS) library preparation and sequencing in the current study.

Here we present a new approach to directly investigate meiotic recombination at the DNA sequence level by combining flow-sorting of pollen nuclei with PicoPLEX single-cell sequencing (Rubicon Genomics). This sequencing approach is based on quasi-random PCR amplification of single-cell genomic DNA and yields a library with dual indexes for limited coverage sequencing. We show that this approach is capable of measuring meiotic recombination and segregation ratios throughout the whole genome of the large genome species barley at megabase resolution by comparing our results obtained through pollen sequencing to genotyping-by-sequencing (GBS) data of a barley DH population.

## Materials and methods

### Plant material and isolation of single pollen nuclei

Pollen grains were collected from a *Hordeum vulgare* L. F_1_ plant derived from a cross between the cultivars “Morex” (♂) and “Barke” (♀) and grown at 20°C during the day (7:00–20:00) and 16°C during the night. Pollen nuclei were isolated and stained as described previously (Dreissig et al., 2015) and sorted using a BD Influx cell sorter (BD Biosciences) into a 384 microwell plate (Applied Biosystems) using the “1.0 drop single” sort mode of the BD FACS software. As a control, we sorted three individual pollen nuclei from the parental genotype “Barke.”

### Single nuclei library preparation and illumina sequencing

Illumina NGS libraries were prepared from 43 individual nuclei using the PicoPLEX DNA-seq kit essentially following the manufacturer's instructions (Rubicon Genomics). After the final amplification reaction with primers containing unique dual barcodes suitable for Illumina NGS, 10 μl aliquots of each library were pooled. The pooled DNA sample was purified using AMPure XP beads (Beckman Coulter Inc.) as described (Rubicon Genomics). The pool was eluted in 30 μl TE (pH 8.0) and size-fractionated using a SYBR-Gold stained 2% agarose gel (Himmelbach et al., 2014). The region of interest (350–1,000 bp) was excised, and the DNA was extracted using the Qiagen MinElute Kit (Himmelbach et al., 2014). The library was characterized using an Agilent 2100 Bioanalyzer (Himmelbach et al., 2014) and quantified by Real-Time PCR as described (Mascher et al., 2013b). After the addition of 8% PhiX DNA as a control, the pooled library was sequenced using the Illumina HiSeq2500 device (rapid run, 1 lane, cBot clustering, 2x 100 cycles paired-end, dual-indexing with 8 cycles per index) according to the manufacturer's instructions. Sequence raw data are available under EMBL ENA accession PRJEB21630.

### Sequence read mapping and genotype analysis

Illumina adapters were trimmed using Cutadapt version 1.12 (Martin, 2011). Trimmed reads were aligned to the barley cv. “Morex” reference genome sequence assembly (Mascher et al., 2017) using BWA-MEM version 0.7.15 (Li, 2013) with default parameters. The resulting SAM files were converted to BAM format with SAMtools (Li et al., 2009). Sorting and detection of optical and PCR duplicates was done with Novosort (http://www.novocraft.com/products/novosort/). SAMtools version 1.3 (Li, 2011) was used for multiple-sample genotype calling at single-nucleotide polymorphism (SNP) sites which were previously ascertained in the “Morex” × “Barke” RIL population using the POPSEQ method (Mascher et al., 2013a). VCF files were imported into the R statistical environment (R Core Team, https://www.r-project.org/contributors.html). Consensus genotypes were derived by aggregating information in 1 Mb bins using functionalities of the R package “data.table” (https://cran.r-project.org/package=data.table). This resulted in a genotype file containing allele information at 1 megabase pair (Mbp) resolution which was used to analyse recombination frequency and segregation distortion.

We used GBS data derived from a “Morex” × “Barke” DH population which was described previously (IBGSC, 2012) for comparison. GBS data were retrieved from https://wheat.pw.usda.gov/ggpages/MxB/. GBS tags were mapped onto the most recent version of the barley reference genome sequence (Mascher et al., 2017) an aggregated in 1 Mbp intervals.

### Recombination analysis based on pollen and a double haploid population

To identify meiotic recombination events in the pollen and double haploid (DH) population, we searched for recombination patterns in each genotype matrix which were indicated by changes from “0” (“Barke” allele) to “2” (“Morex” allele) or vice versa. To count recombination events, we conducted a text search for patterns indicating recombination events (e.g., 0 → 0 → 0 → 2 → 2 → 2). We manually curated the genotype files by removing markers showing a high frequency of double crossovers (e.g., 0 → 2 → 0), which were considered genotyping errors (Salome et al., 2012). To map the approximate position of recombination events onto the physical map of the barley genome, a 5-Mbp sliding window approach was used to scan along each chromosome searching for allele changes from “0” to “2” and vice versa. We then calculated recombination frequency in cM/Mbp [cM = 100^*^(# of recombinations/#total)] along each chromosome by counting the number of recombination events in 5-Mbp sliding windows relative to the total number of samples. To analyse crossover interference, we extracted all samples showing more than two recombination events on a given chromosome and calculated the physical distance (Mbp) between nearby recombination events. To determine the effect of crossover interference, we used the crossover distribution analyser (CODA) software (Gauthier et al., 2011) which compares observed inter-crossover distances against a simulated gamma model to calculate *nu*. A value of *nu* = 1 indicates no interference, *nu* < 1 indicates negative interference, and *nu* > 1 indicates positive interference. Genotype data are available as Supplementary File 1.

### Analysis of segregation distortion in pollen and double haploid population

Segregation distortion was analyzed by calculating average allele frequencies in 10 Mbp sliding windows along each chromosome of both populations. Markers with >50% missing data were removed from the analysis. To test for significant deviation from the expected segregation ratio of 1:1 of each parental allele, we conducted a χ^2^-test between expected and observed allele frequencies. Segregation distortion regions (SDR) were identified by a significant deviation from the expected ratio of 1:1 (*P* < 0.05).

## Results

### Sequencing of individual pollen nuclei

**Figure 1:**
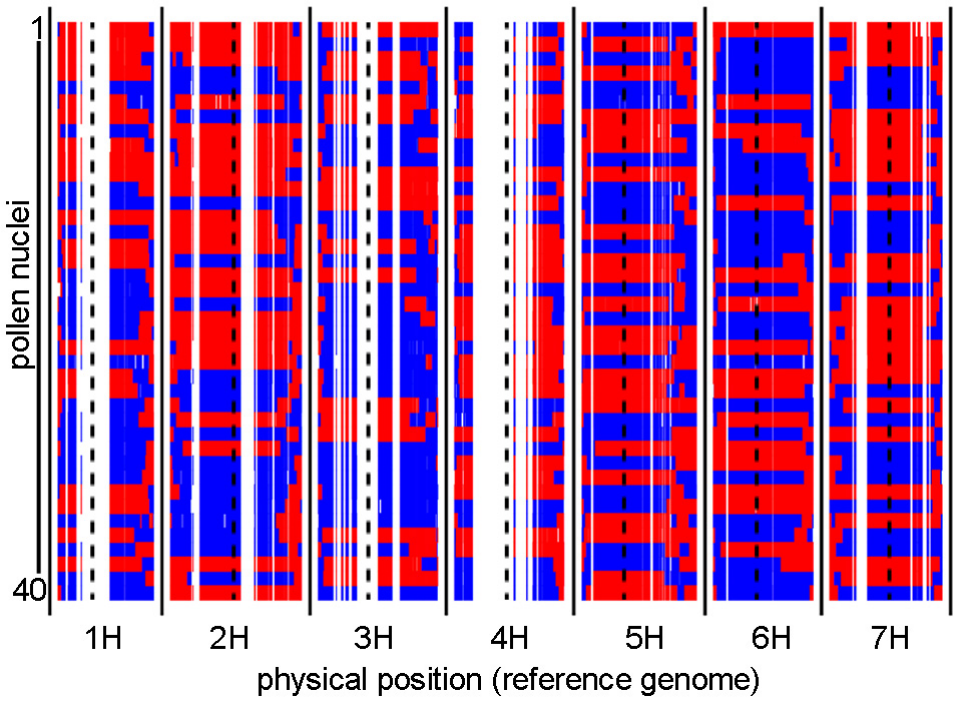
Graphical genotypes of individual pollen nuclei revealed by single-cell genome sequencing. Recombination events were detected in 40 individual pollen nuclei. The two parental barley genotypes are shown in red (“Morex”) and blue (“Barke”). Consensus genotypes were mapped to the physical reference genome of barley at 1 Mbp resolution. Centromere positions are indicated by dashed black lines. White gaps which consistently occur in all samples are regions where no genetic polymorphisms exist between “Morex” and “Barke.”

To identify recombination events, we first sequenced the genomes of individual haploid pollen nuclei. Toward this purpose, we utilized our previously established approach for pollen nuclei isolation (Dreissig et al., 2015) combined with PicoPLEX single-cell DNA amplification and NGS library preparation. A total of 40 pollen nuclei derived from a single “Morex” (♂) x “Barke” (♀) F_1_ plant were subjected to PicoPLEX sequencing. As a control, pollen nuclei obtained from the parental genotype “Barke” were used. The initial DNA amplification via quasi-random priming yielded an average fragment size of 933 bp. No amplification was detected in the negative control which indicates that the amount of DNA contamination was below the level of detection. Sequencing the 40 pollen nuclei on the Illumina HiSeq 2500 platform yielded between 2.7 million and 11.6 million (mean: 5.9 million) reads per sample, corresponding to an average read depth of 0.1x per haploid nucleus. Reads were mapped to the reference genome assembly of cv. “Morex” (Mascher et al., 2017) and genotypes were called at single-nucleotide polymorphism (SNP) sites known to segregate in the “Morex” × “Barke” population (Mascher et al., 2013a). Consensus genotypes were derived by aggregating SNP information in 1 Mbp bins based on the reference genome. Figure 1 shows the graphical genotypes of the 40 pollen nuclei at 1 Mbp resolution.

### Comparing the recombination landscape of barley pollen and DH plants

Based on cytological analyses (Sybenga, 1966; Phillips et al., 2013; Aliyeva-Schnorr et al., 2015) and molecular analyses of segregating populations (Künzel et al., 2000; IBGSC, 2012; Phillips et al., 2015), the recombination landscape of barley is characterized by elevated recombination frequencies in distal chromosome regions and strongly reduced recombination in (peri-)centromeric regions. In order to overcome the resolution limit of cytological analyses, we attempted to investigate the recombination landscape of barley directly at the DNA sequence level by sequencing individual pollen nuclei.

**Figure 2:**
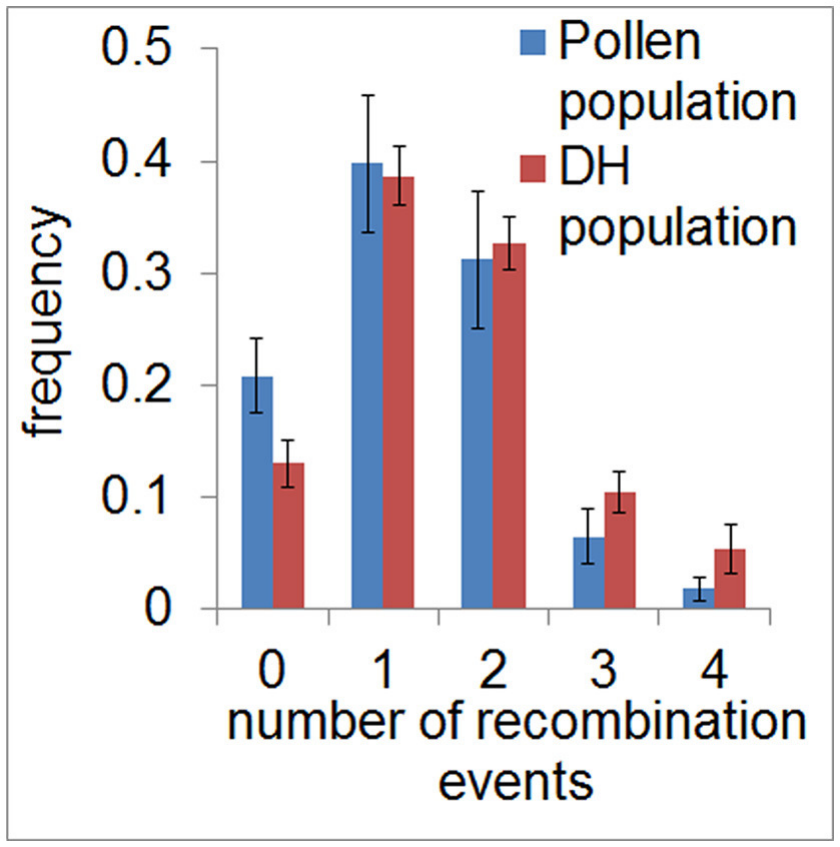
Frequency of recombination events in pollen and DH plants. Relative frequency of the average number of recombination events per chromosome is shown for the pollen (blue) and DH population (red) in classes ranging from 0 to 4. Error bars represent the standard deviations based on measurements conducted on all seven barley chromosomes.

To assess the recombination landscape of barley pollen compared to DH plants, we first counted the number of recombination events in each sample in both populations. We measured a total of 380 recombination events in the population of 40 haploid pollen nuclei (average of 9.5 per pollen nucleus, SE = 0.38) and 974 recombination events in the DH population composed of 89 plants (average of 10.9 per DH plant, SE = 0.3). Predominantly, we detected one or two recombination events per chromosome in both populations with 38.7–39.8% of samples showing one recombination event and 31.1–32.6% of samples showing two recombination events. The number of recombination events, which was ranging from zero to four per chromosome, was found to be similar between pollen and DH population (χ^2^-goodness of fit test, *P* > 0.99978) (Figure 2). The occurrence of chromatids apparently lacking any recombination event detected by SNPs (13–20%) seems to be the same as in an *Arabidopsis* data set described by Salome et al. (2012). Consequently, recombination frequency was found to be similar in barley pollen compared to whole DH plants.

**Figure 3:**
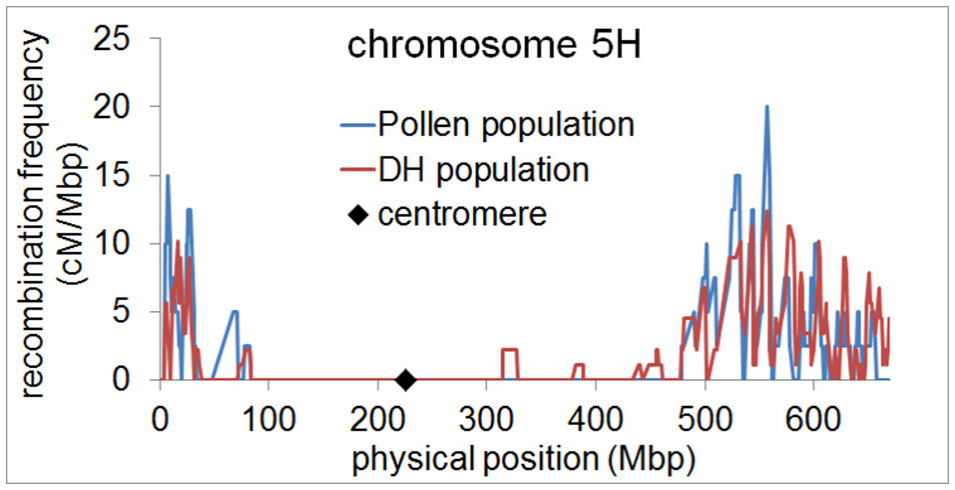
Elevated recombination frequencies in distal regions of barley chromosome 5H. Recombination frequency in pollen (blue) and DH plants (red) was calculated in 5 Mbp sliding windows along chromosome 5H and plotted along the physical map. The position of the centromere is marked by a black diamond.

Since the number of recombination events per chromosome was highly similar between the pollen population and the DH population, we then examined whether the genome wide distribution of recombination events differed between both populations. We measured recombination frequencies along all chromosomes of barley using a 5 Mbp sliding window approach. In both populations, we found elevated recombination frequencies in distal regions of all chromosomes and almost no recombination in (peri-)centromeric regions (Figure 3, Supplementary files 2–7). This observation is in agreement with previous studies showing a skewed distribution of recombination events toward distal chromosome regions in barley (Künzel, 1982; Linde-Laursen, 1982; Künzel et al., 2000; Phillips et al., 2013; Baker et al., 2014; Dreissig et al., 2015). It also shows that there is no different positioning of recombination events in pollen, i.e., in (peri-)centromeric regions. These regions were shown to harbor essential genes encoding proteins for basic cellular functions such as translation and photosynthesis (Mascher et al., 2017). It could therefore be reasoned that (peri-)centromeric recombination events could theoretically be absent in DH plants due to selection against housekeeping gene-encoding (peri-)centromeric sites of recombination which would disrupt linkage between essential genes.

**Figure 4:**
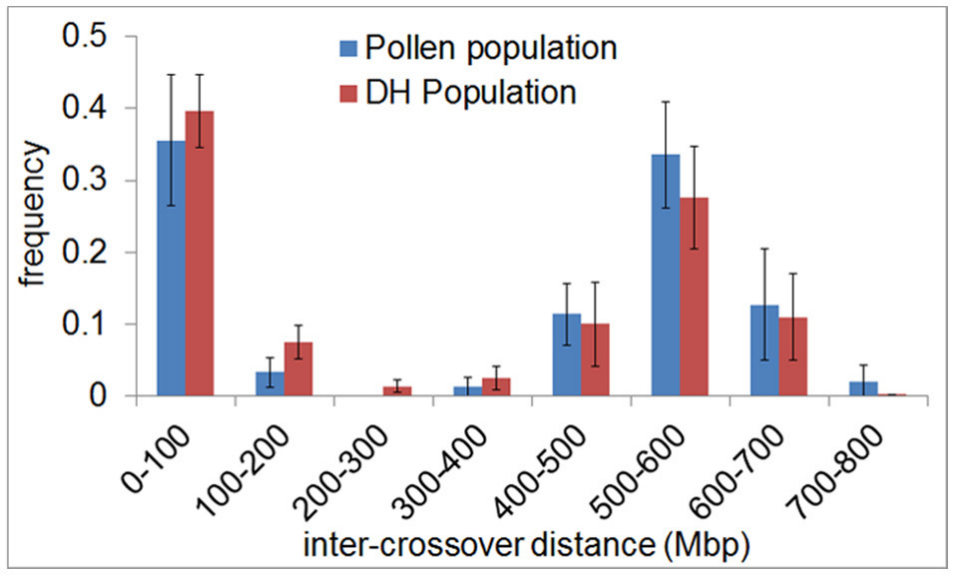
Inter-crossover distance reveals positive crossover interference and supports the existence of two crossover classes in barley. The frequency of the distance between crossovers on the same chromatid (inter-crossover distance) in pollen (blue) and DH plants (red) was determined in 100 Mbp classes ranging from < 100 to >700 Mbp. The relative frequency of nearby crossovers present in each class was plotted. Error bars represent the standard deviation based on measurements conducted on all seven barley chromosomes.

**Figure 5:**
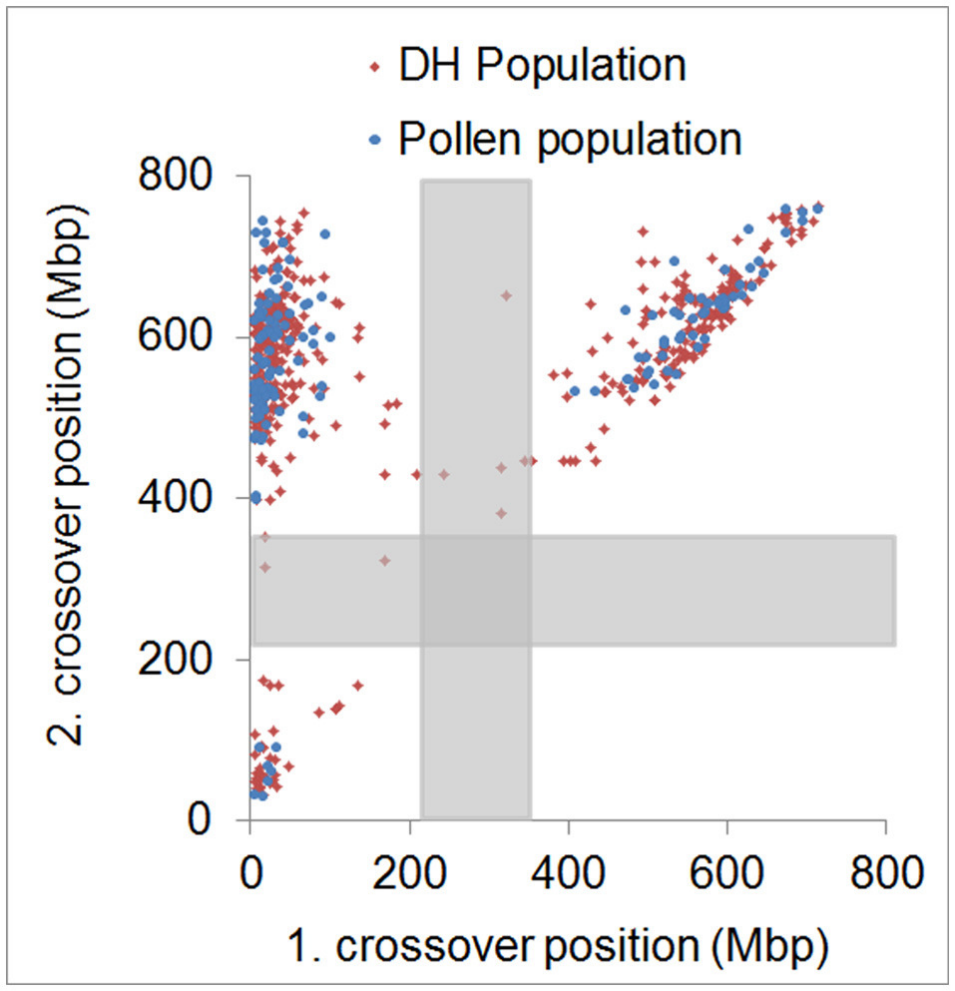
Physical distribution of first and second crossover positions. Physical positions of first and second crossover event for all samples showing more than two crossovers in the pollen (blue) and DH (red) population. Approximate centromeric regions are marked by gray boxes. Strong physical interference is shown by dots accumulated in the top left quarter. Weak physical interference is shown by dots accumulated in the bottom left and top right quarter.

In agreement with the predominantly distal positioning of recombination events in both populations, we found positive crossover interference indicated by 48.9–59.8% of recombination events being separated by more than 400 Mbp (range = 402–729 Mbp) over a chromosome size ranging from 558 to 767 Mbp. Interestingly, 35.6–39.6% of recombination events were separated by less than 100 Mbp (range = 10–98 Mbp) (Figure 4). The smallest distance between two recombination events was 10 Mbp which corresponds to ~1.5% of the chromosome. We conducted a crossover interference analysis (gamma model; measured in *nu*) to determine the strength of interference (Gauthier et al., 2011). A value of *nu* = 1 indicates no interference, *nu* < 1 indicates negative interference, and *nu* > 1 indicates positive interference. Due to the low number of chromosomes showing at least two recombination events, we did not analyse chromosomes separately, but pooled data from all seven barley chromosomes. Positive interference values of *nu* = 4.76 and 3.02 were detected in DH and pollen populations, respectively. In addition, we split all recombination events into two groups with < 100 or >400 Mbp distance between two events. When both groups were analyzed separately, we found weaker interference values for recombination events less than 100 Mbp apart (*nu* = 2.336 for pollen and *nu* = 2.202 for DH population) and stronger interference values when more than 400 Mbp apart (*nu* = 8.511 for pollen and *nu* = 8.199 for DH population). These patterns might be attributed to interference sensitive and less sensitive crossovers, i.e., class I and class II crossover. We then tested whether recombination events separated by less than 100 Mbp were confined to specific chromosomal regions or distributed randomly by plotting the physical positions of multiple recombination events on the same chromosome against themselves (Figure 5). All recombination events separated by less than 100 Mbp were strictly confined to distal regions, which corresponds to the accumulation of dots in the bottom left and top right quarters of Figure 5. Recombination events separated by more than 400 Mbp were located on different arms (dots in the top left quarter of Figure 5). Our data show that crossover interference is positive in barley. However, a substantial proportion of recombination events is separated by less than 100 Mbp which supports the existence of class I and class II crossovers in barley.

### Segregation distortion is high in DH plants, but almost absent in pollen

Segregation distortion is defined as the preferential transmission of one allele over the other, which results in a statistically significant deviation from an expected Mendelian segregation ratio of 1:1. We asked whether the extent of segregation distortion differs between pollen and DH plants. Our hypothesis was that segregation distortion would be substantially lower in pollen because of the absence of any selective pressure which might arise during pollen tube growth, fertilization, hybrid compatibility, and plant development. We expected the opposite in the DH population because of selective pressure during microspore culture, embryo development, plant regeneration, and spontaneous diploidization. It is important to note that the DH population which was genotyped and provided by the IBGSC (2012) consisted of spontaneously diploidized plants only.

**Figure 6:**
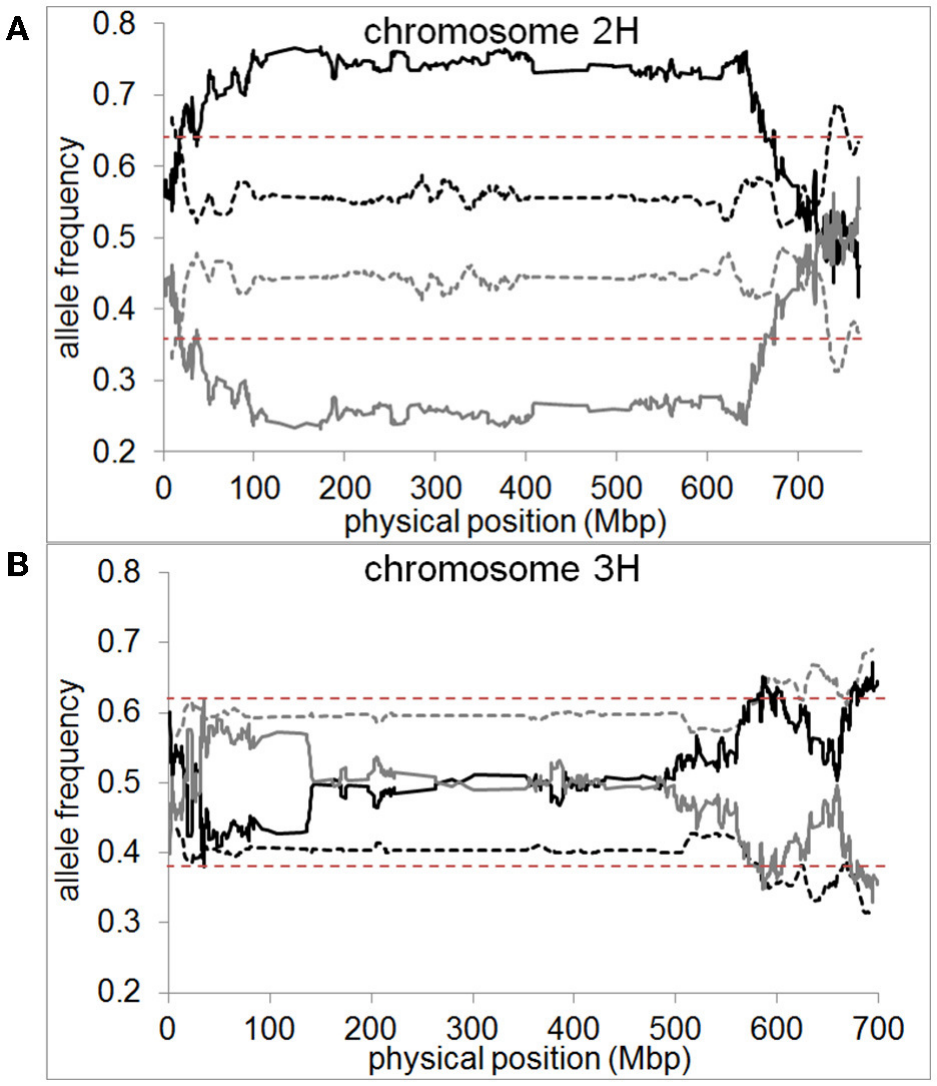
Segregation distortion is almost absent if measured in pollen but abundant in DH plants. Allele frequencies for “Morex” (black) and “Barke” (gray) measured in pollen (dashed line) and DH plants (straight line) are shown as 10 Mbp moving averages for **(A)** chromosome 2H and **(B)** chromosome 3H of barley. Dashed red lines represent the significance threshold of distorted segregation ratios (χ^2^-test, *P* < 0.05). Pollen or DH allele frequencies above the significance threshold mark genomic regions of distorted segregation ratios.

In the pollen population, we found normal segregation ratios for almost all chromosomal regions (Supplementary files 8–12). The exceptions were one region on chromosome 2H located at 736–752 Mbp and two regions on chromosome 3H located at 634–642 Mbp and 682–695 Mbp (Figure 6). These regions only amount to 2 and 3% of chromosome 2H and 3H, respectively. In both cases, these SDRs were located in high recombining regions of the chromosome allowing them to remain small and not cause distorted segregation of a larger part of the chromosome through linkage (Supplementary file 13). In contrast, in the DH population, a high proportion of large chromosomal regions were affected by segregation distortion. We detected a total of 15 SDRs distributed across all chromosomes which varied in size ranging from 0.01 up to 87.3% of the chromosome. Major SDRs, varying from 72.6 up to 87.3% of the chromosome, were found on chromosome 1H, 2H, 5H, and 7H (Figure 6A, Supplementary files 8, 10, 12). In addition to these major SDRs, we detected 11 minor SDRs which varied in size ranging from 0.01 up to 5% of the chromosome (Figure 6B, Supplementary files 8, 10–12). Interestingly, we did not detect the same SDRs on chromosome 2H and 3H in the pollen population as in the DH population which indicates different selective pressures acting on these loci. For example, in the DH population, two regions of chromosome 3H (571.6–606.6 Mbp and 672.2–698.3 Mbp) exhibited higher transmission of the “Morex” allele whereas, in the pollen population, two regions of the chromosome (634–642 Mbp and 682–695 Mbp) exhibited higher transmission of the “Barke” allele (Figure 6B). This example shows that under varying conditions (e.g., pollen development vs. DH production) not only different regions can be selected, but also different parental alleles can be preferentially transmitted.

Hence, our results show that segregation distortion is almost absent in pollen grains which supports the conclusion that meiosis alone is not the main cause of this phenomenon. On the contrary, segregation distortion was found for nearly half of the entire genome (49.9%) in barley DH plants. We conclude that selective pressure during microspore culture, embryo development, plant regeneration, and diploidization is the most likely cause for segregation distortion in DH plants.

## Discussion

The main conclusion of the present study is that the recombination landscape of barley pollen and DH plants does not differ in frequency or positioning of recombination events, yet segregation distortion is almost absent in pollen grains whereas it is detectable to a large extent in DH plants likely caused by selection during DH production. In addition, we present recombination measurements which support the existence of class I and class II crossovers in barley. We demonstrate that our approach for single pollen nuclei sequencing is suitable to directly investigate the recombination landscape of barley at the molecular level in an unbiased way.

### Pollen sequencing as a robust approach to directly measure recombination at megabase resolution in barley

We sought to analyse recombination in pollen and DH plants separately to test if the typical recombination pattern found in segregating populations of barley, characterized by a predominantly distal positioning of recombination events, is caused by selection against (peri-)centromeric recombination events or reflects the real outcome of meiosis. The low recombining regions of the barley genome were previously shown to constrain gene diversity (IBGSC, 2012; Baker et al., 2014). This phenomenon is widespread in nature and is most likely caused by a combination of selective sweeps via fixation of advantageous alleles and background selection against deleterious mutations (Hill and Robertson, 1966; Smith and Haigh, 1974; Hudson, 1994; Wright et al., 2006). Furthermore, it was recently shown that essential genes involved in translation and photosynthesis reside in (peri-)centromeric low-recombining regions of the barley genome (Mascher et al., 2017). It could thus be argued that recombination events in low-recombining regions would break linkage between advantageous alleles and therefore be selected against. In pollen, however, these recombination events could still be present due to the absence of selective pressure which certainly arises during pollen tube growth, fertilization, and plant development (Pedersen, 1988; Sarigorla et al., 1992; Walsh and Charlesworth, 1992).

Our data show that the recombination landscape of barley, characterized by elevated recombination frequencies in distal regions (Figure 3), is truly the outcome of meiosis and not a result of postmeiotic selection against (peri-)centromeric recombination events. This is in agreement with previous cytogenetic studies taking direct recombination measurements by means of scoring MHL3 immunostaining foci or chiasmata (Bennett et al., 1973; Phillips et al., 2013). However, it was of interest for us to test if these observations reveal the same recombination landscape as by sequencing of pollen nuclei. The direct sequencing of pollen nuclei, through the approach presented in this study, offers a much higher resolution in detecting the positions of recombination events (i.e., 1 Mbp, approximately 0.2% of the smallest barley chromosome) compared to the mapping of MLH3 fluorescence foci during meiotic prophase by structured illumination microscopy (Phillips et al., 2013). Compared to chiasmata counts performed in a variety of barley genotypes, the average number of recombination events detected in our study seems to be lower (Gale et al., 1970; Bennett et al., 1973; Colas et al., 2016). If it holds true that all cytologically defined chiasmata represent genetic exchanges between homologous chromosomes, we cannot exclude that certain recombination events are missing in our data sets. On the other hand, we measured similar recombination frequencies in pollen and DH plants while both populations were genotyped by two different methods, i.e., single-cell sequencing vs. genotyping-by-sequencing of DH plants. Furthermore, both approaches are based on haploid male gametes where only one of the four possible meiotic products, i.e., chromatids, is present. Hence, as evident from *Arabidopsis* tetrad analysis where all four chromatids are analyzed (Lu et al., 2012; Wijnker et al., 2013), it is possible for a haploid pollen nucleus to contain the exact chromatid that did not undergo meiotic recombination. It is therefore unlikely that single cell sequencing accounts for missing recombination events. It could also be argued that these differences reflect genotypic variations or environmental effects as such were shown in many cases (Sall et al., 1990; Bauer et al., 2013; Phillips et al., 2015; Sidhu et al., 2015; Ziolkowski et al., 2015, 2017).

We detected positive crossover interference in both pollen and DH plants, which is in agreement with the primarily distal positioning of recombination events. Previously, Phillips et al. (2013) reported for barley that 34–38% of crossovers are < 20% of chromosome length apart and the majority of crossovers are >70% apart which results in a bimodal distribution of inter-crossover distances. Here, we found 36.8–40.4% of crossovers separated by less than 100 Mbp (approximately 15% of chromosome length) and 48.3–57.4% separated by more than 400 Mbp (approximately 60% of chromosome length) reflecting a similar bimodal distribution of inter-crossover distances (Figure 4). The minimum inter-crossover distance found in our study was 10 Mbp which refers to 1.5% of the corresponding chromosome. We quantified crossover interference strength (gamma model; measured in *nu*) in the pollen and DH population. We detected positive physical interference between crossovers in both pollen (*nu* = 3.02) and DH population (*nu* = 4.76). These interference values are higher than those previously reported for the barley cultivar “Morex,” which was at *nu* = 1.58 (Phillips et al., 2013). However, Higgins et al. (2014) argued that crossover interference might actually be stronger than estimated by Phillips et al. (2013) because the relative separation of MLH3 foci was measured when synapsis of chromosomes was completed and not at the exact time point when crossover designation took place during synapsis. Our data, which are based on scoring crossovers at the sequence level, support this hypothesis by showing stronger crossover interference values for barley.

The existence of two crossover classes, namely class I for interference-sensitive crossovers and class II for interference-insensitive crossovers, was shown in *S. cerevisiae* and *A. thaliana* mutants being defective for core components involved in class I crossover formation (Börner et al., 2004; Higgins et al., 2004). In these mutants, 15% of crossovers of the wild-type level were still formed, which indicates the existence of an alternative class II pathway. However, the presence of two crossover classes has not been confirmed experimentally in barley yet although increasing evidence supports their existence (Phillips et al., 2013, 2015). In our study, the occurrence of recombination events separated by < 100 or >400 Mbp supports the existence of interference-sensitive and less sensitive crossovers, i.e., class I and class II. However, it remains a matter of speculation why nearby crossovers are strictly confined to distal regions and do not span (peri-)centromeric regions. There is a well-known correlation between low-recombining (peri-)centromeric regions and certain histone modifications in barley, i.e., histone H3K9me2, H3K9me3, H3K27me1, and H3K27me2, as shown by chromatin immunoprecipitation (ChIP) sequencing in barley seedlings (Baker et al., 2015). Furthermore, it was shown in *Arabidopsis* that DNA methylation restricts crossovers in centromeric regions and that crossover hot spots are associated with active chromatin modifications such as H2A.Z and H3K4me3 (Yelina et al., 2012; Choi et al., 2013). It could therefore be argued that by changing specific DNA or histone modifications, crossover positioning could be manipulated to increase genetic recombination in (peri-)centromeric regions in crops such as barley.

### Comparison of segregation distortion in pollen and DH plants

Segregation distortion is a widespread phenomenon in plant populations characterized by a deviation from the expected Mendelian segregation ratio. For plant breeders, it presents a problem as it has an effect on allele frequencies and can reduce the chances of obtaining specific combinations of alleles. Double haploid technology has developed into one of the most important methods for plant breeders to accelerate the otherwise lengthy process of obtaining homozygous genotypes (Germana, 2011). The disadvantage of this technology is that it is accompanied by segregation distortion to a very high extent in many genotypes and species (Xu et al., 1997; Taylor and Ingvarsson, 2003; Bélanger et al., 2016a). Segregation distortion during DH production appears to be caused by selective pressure acting upon certain loci or genomic regions. Selective pressure might arise during microspore culture, embryogenesis, plant regeneration, and spontaneous diploidization of haploid plants. Bélanger et al. (2016b) have shown that segregation distortion in barley arises predominantly during embryogenesis and plant regeneration.

In the current study, we hypothesized that segregation distortion would be low if measured in pollen grains due to the absence of selective pressure. Our data show that only three small chromosomal regions show distorted segregation ratios in pollen, amounting to 0.8% of the genome, whereas nearly 50% of the genome shows distorted segregation ratios in DH plants. This suggests that segregation distortion is not a direct outcome of meiosis but a product of selection acting at different developmental stages. Compared to Bélanger et al. (2016b) who detected no segregation distortion in immature pollen, we found one region on chromosome 2H and two regions on chromosome 3H with distorted segregation rations in mature pollen. It can be speculated that these regions might play a role in pollen development and therefore show distorted segregation. Furthermore, environmental conditions, e.g., heat stress (Frova and Sari-Gorla, 1994) or higher nutrient levels in the soil (Martin et al., 2017) can have an effect on segregation ratios in pollen, although our experiment did not involve any stress treatment.

Further improvements in protocols and decreases in the price of sequencing should enable the application of single pollen sequencing as a novel prediction tool in research and plant breeding in a wide range of species.

## Author contributions

SD isolated pollen nuclei, conducted flow-sorting, analyzed the data, and wrote the manuscript. JF conducted flow-sorting, contributed to the manuscript and edited the manuscript. AHi conducted PicoPLEX single-cell sequencing, contributed to the manuscript and edited the manuscript. MM processed all raw data, analyzed the data, contributed to the manuscript and edited the manuscript. AHo conceptualized the experiments, supervised the analyses, contributed to the manuscript and edited the manuscript. All authors read and approved the final version of this manuscript.

### Conflict of interest statement

The authors declare that the research was conducted in the absence of any commercial or financial relationships that could be construed as a potential conflict of interest.
