# Supplementary figures and images for: Sequencing of Single Pollen Nuclei Reveals Meiotic Recombination Events at Megabase Resolution and Circumvents Segregation Distortion Caused by Postmeiotic Processes
Source: Front Plant Sci. 2017 Sep 26;8:1620. doi: 10.3389/fpls.2017.01620 (PMC5623100; doi:10.3389/fpls.2017.01620)

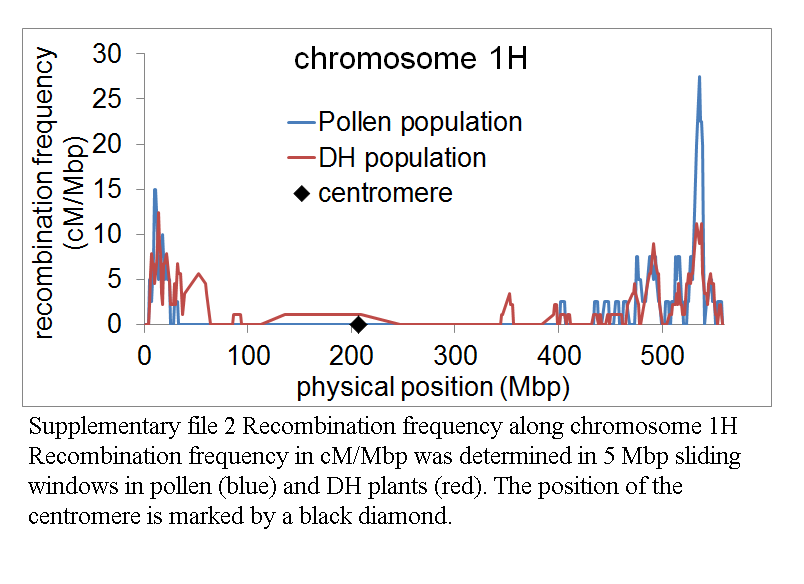

Supplement: Supplementary file 2 [file Image1.TIF]

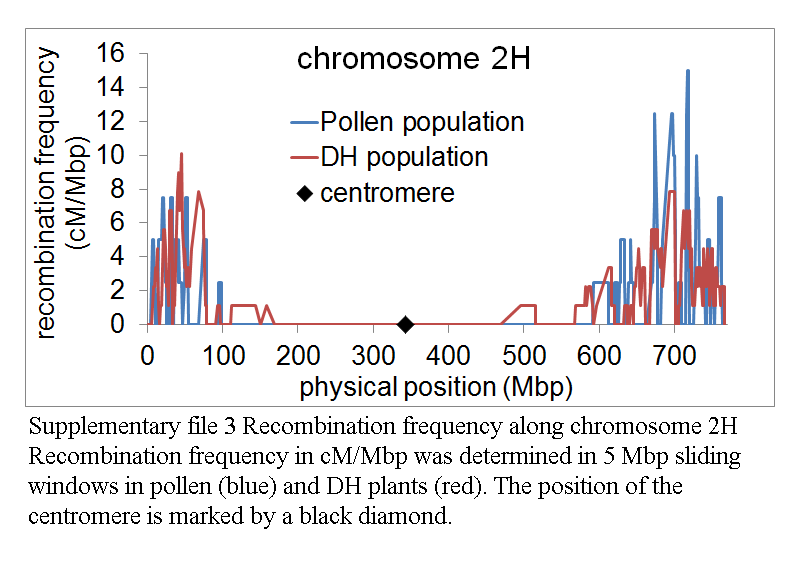

Supplement: Supplementary file 3 [file Image2.TIF]

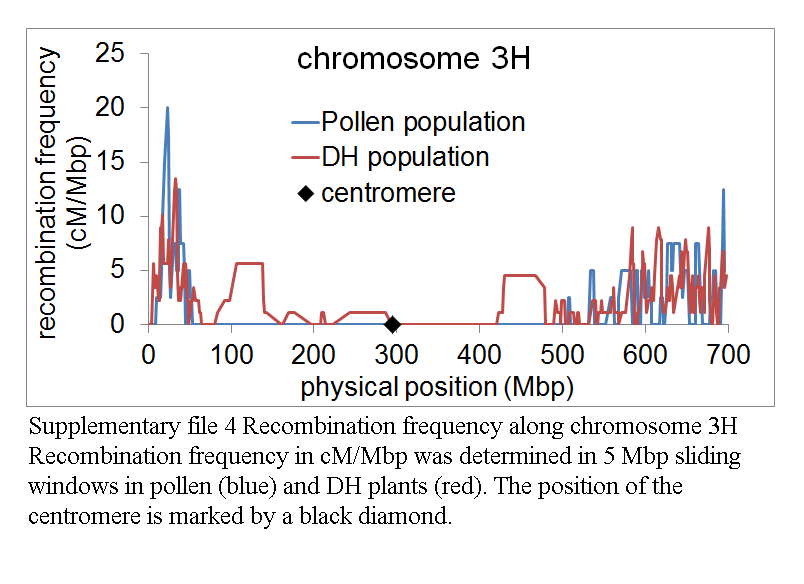

Supplement: Supplementary file 4 [file Image3.TIF]

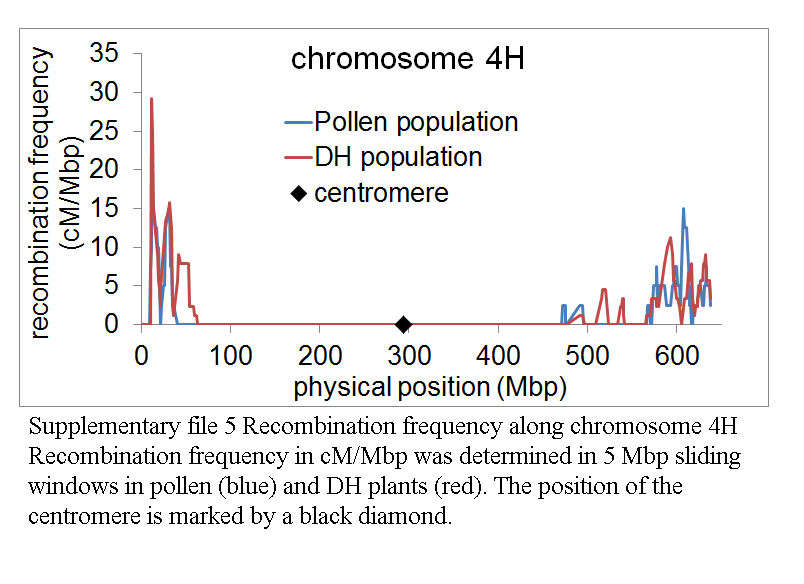

Supplement: Supplementary file 5 [file Image4.TIF]

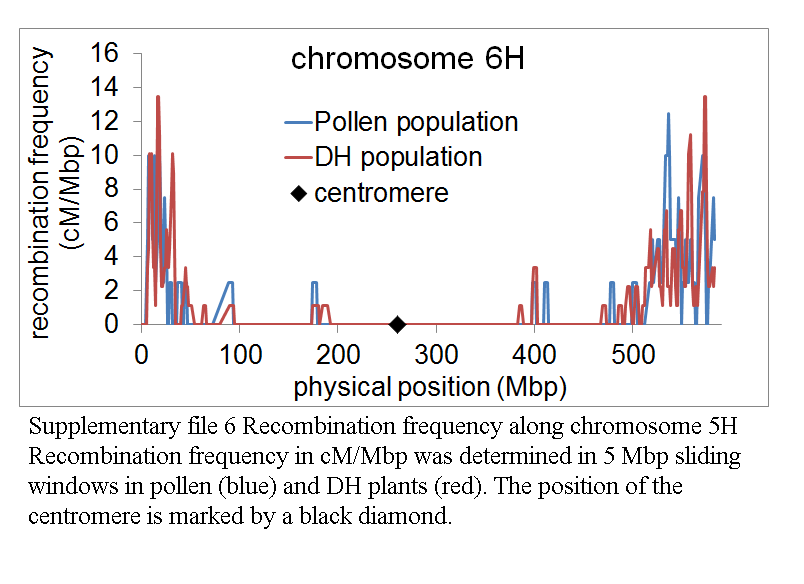

Supplement: Supplementary file 6 [file Image5.TIF]

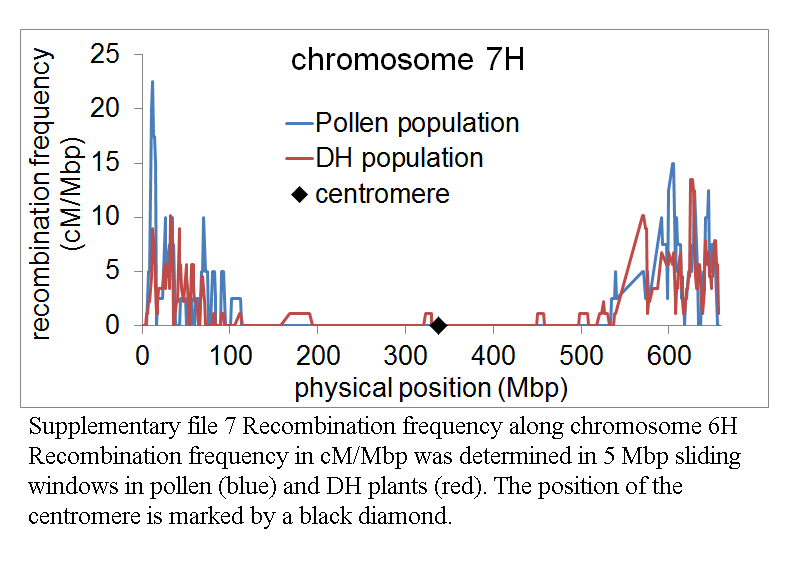

Supplement: Supplementary file 7 [file Image6.TIF]

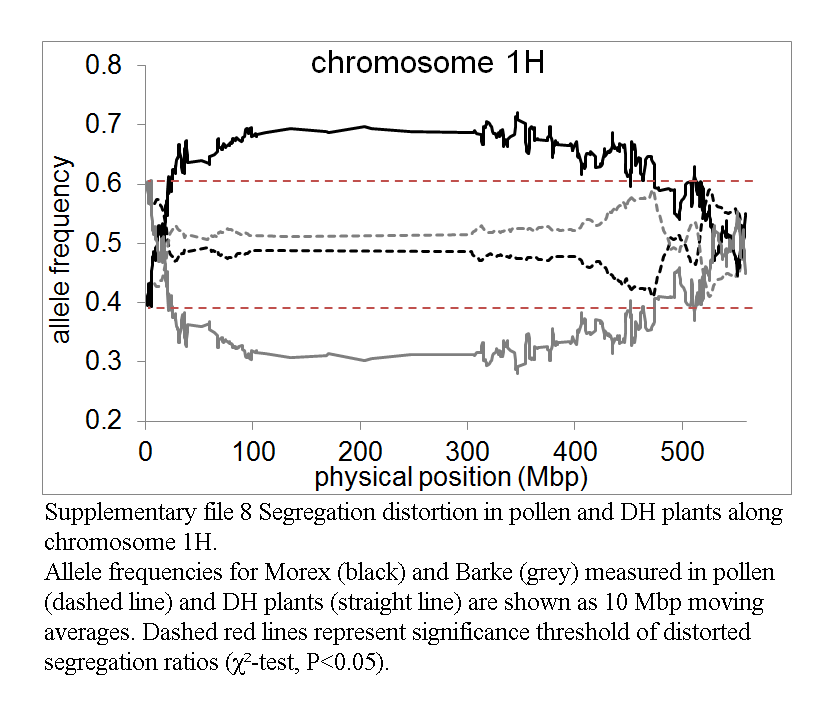

Supplement: Supplementary file 8 [file Image7.TIF]

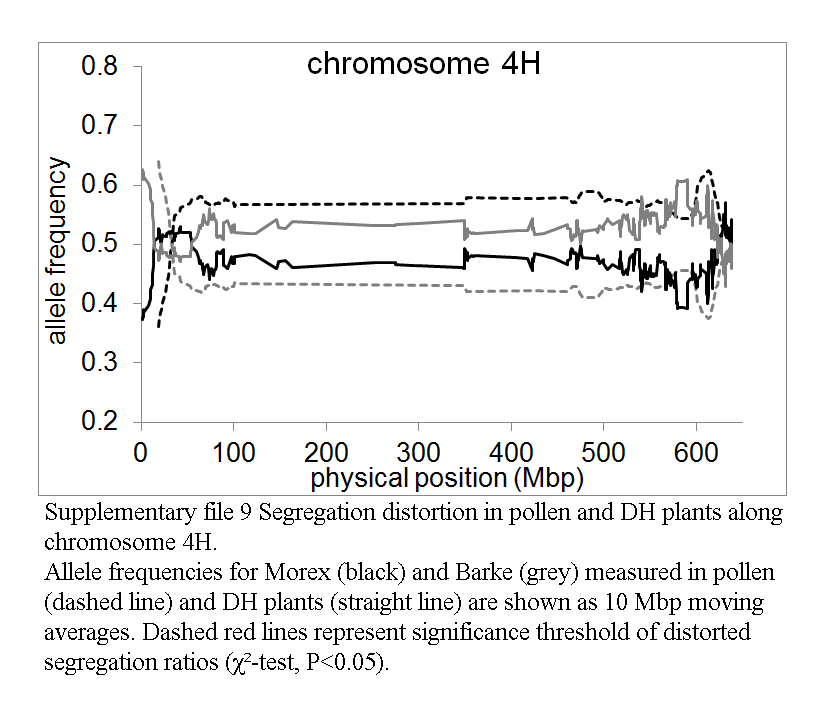

Supplement: Supplementary file 9 [file Image8.TIF]

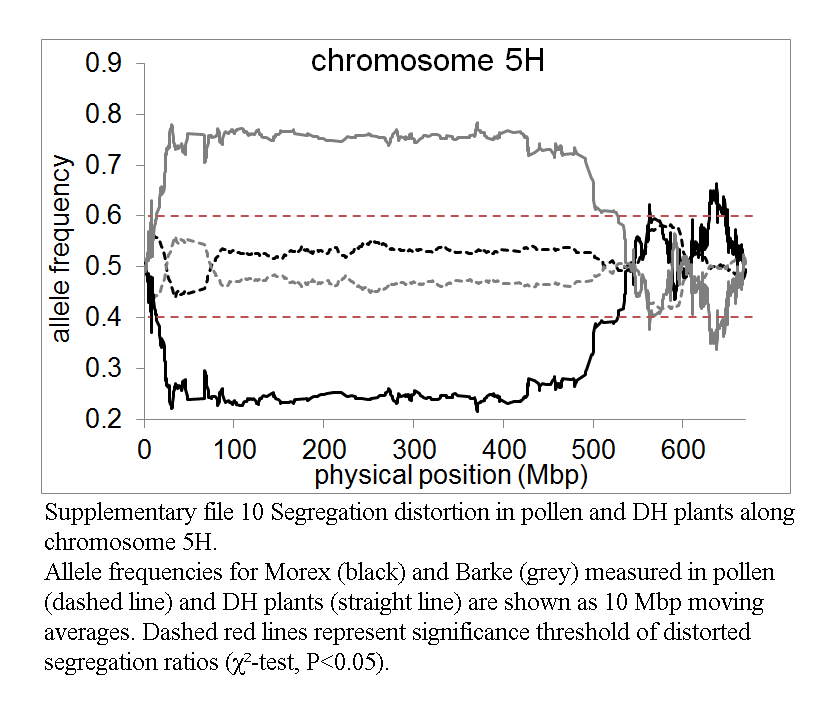

Supplement: Supplementary file 10 [file Image9.TIF]

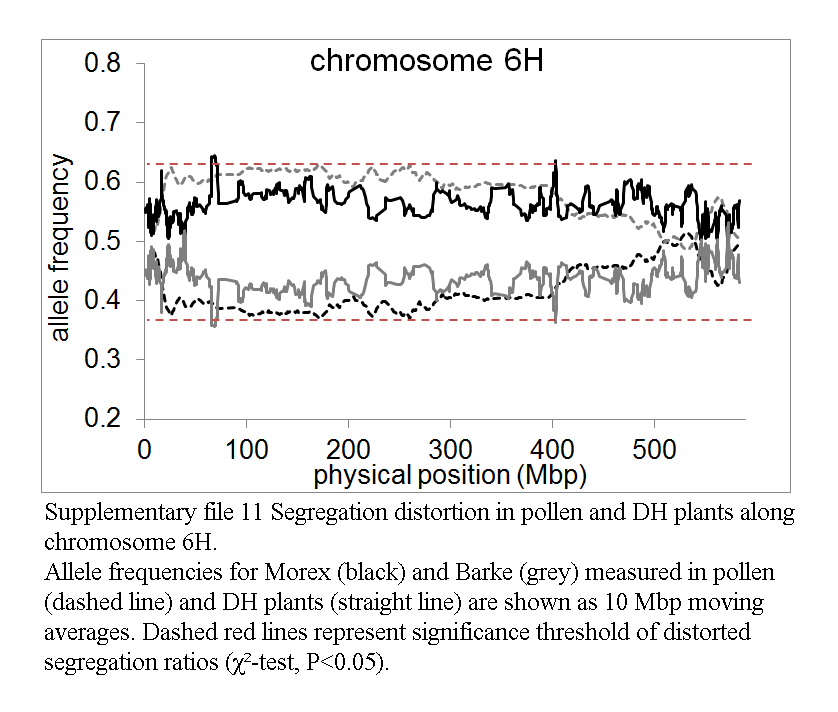

Supplement: Supplementary file 11 [file Image10.TIF]

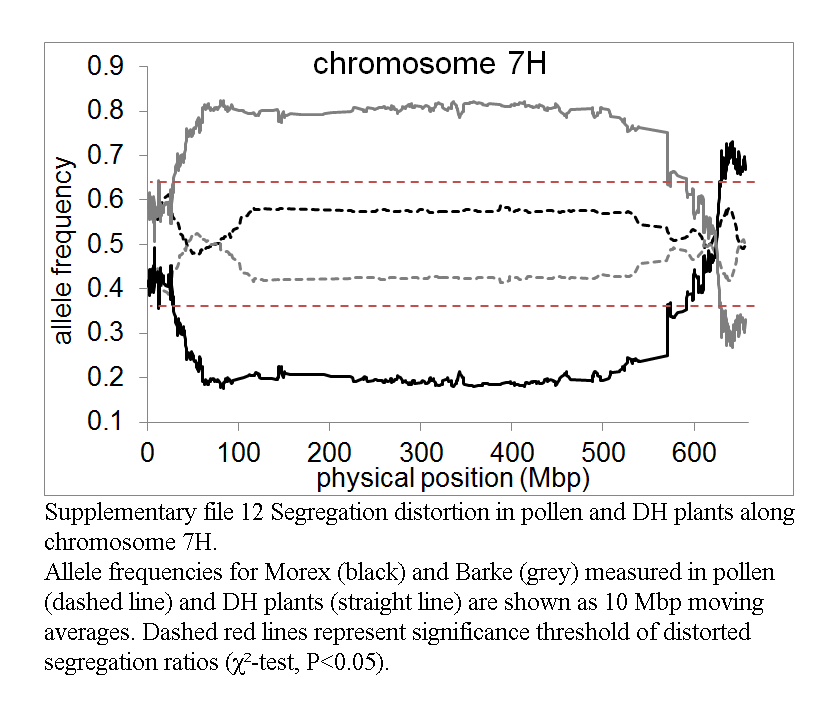

Supplement: Supplementary file 12 [file Image11.TIF]

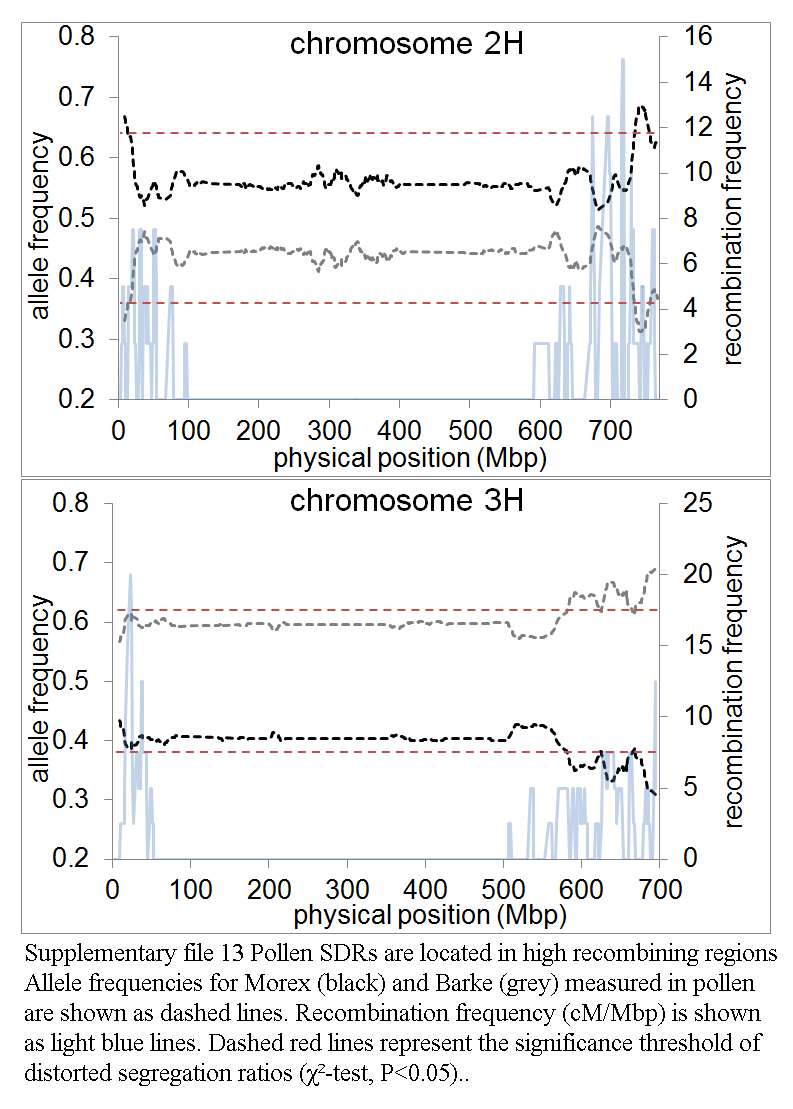

Supplement: Supplementary file 13 [file Image12.TIF]
